# A Cross-Sectional Exploratory Study of Endothelial Biomarkers in Non-Obese Women with and Without Polyendocrine Metabolic Ovarian Syndrome

**DOI:** 10.3390/ijms27177661

**Published:** 2026-08-26

**Authors:** Manjula Nandakumar, Sana Waris, Thozhukat Sathyapalan, Alexandra E. Butler, Stephen L. Atkin

**Affiliations:** 1 Research Department, Royal College of Surgeons in Ireland, Medical University in Bahrain, Adliya P.O. Box 15503, Bahrainabutler@rcsi.com (A.E.B.); satkin@rcsi.com (S.L.A.); 2 Academic Endocrinology, Diabetes and Metabolism, Hull York Medical School, Hull HU6 7RX, UK

**Keywords:** polyendocrine metabolic ovary syndrome, PMOS, endothelium, cardiovascular risk, biomarkers, VEGF, vitamin D

## Abstract

Endothelial dysfunction has been reported in polyendocrine metabolic ovarian syndrome (PMOS) and is associated with obesity. We hypothesised that circulating biomarkers of endothelial dysfunction would not differ between non-obese women with PMOS and age- and BMI-matched controls in the absence of major between-group differences in insulin resistance (IR), systemic inflammation or vitamin D status. Plasma concentrations of 20 endothelial-associated proteins were measured using Slow Off-rate Modified Aptamer (SOMAscan) technology in 29 non-obese women with PMOS and 29 age- and BMI-matched controls. Women with PMOS had higher Ferriman–Gallwey scores, free androgen index (FAI) and anti-Müllerian hormone (AMH), whereas HOMA-IR, C-reactive protein (CRP), vitamin D metabolites and endothelial-associated biomarkers did not differ significantly between groups. Within the PMOS cohort, FAI was strongly positively associated with E-selectin (Pearson *r* = 0.718, *p* < 0.001, FDR-adjusted *q* = 0.027), and HOMA-IR was positively associated with ICAM-2 (*r* = 0.670, *p* = 0.001, *q* = 0.046). The FAI–E-selectin association remained robust in Spearman sensitivity analysis (ρ = 0.720, *p* < 0.001, *q* = 0.009), whereas the HOMA-IR–ICAM-2 association remained nominally significant (ρ = 0.579, *p* = 0.007) but not after FDR correction. A positive association between 24,25(OH)_2_D_3_ and tumour necrosis factor (TNF) was observed using Pearson correlation (*r* = 0.648, *p* = 0.002, *q* = 0.042), but was not retained after FDR correction in Spearman sensitivity analysis. Thus, non-obese women with PMOS did not demonstrate a generalised alteration in circulating endothelial biomarkers compared with matched controls. Hyperandrogenism, in particular, was robustly associated with endothelial activation, whereas associations with IR were less consistent and vitamin D metabolites showed no robust relationship with the endothelial biomarker profile. Collectively, the findings suggest that variation in endothelial activation in non-obese PMOS is more closely associated with metabolic dysfunction and androgen excess than with circulating vitamin D status, while the cross-sectional design precludes inference regarding causality.

## 1. Introduction

Polyendocrine metabolic ovarian syndrome (PMOS), formerly termed polycystic ovary syndrome (PCOS), is the most common endocrine disorder affecting women of reproductive age, with an estimated global prevalence of 10–13% [1]. In addition to its reproductive manifestations, PMOS is associated with metabolic abnormalities including insulin resistance (IR), type 2 diabetes and hypertension [2,3]. Whether these abnormalities translate into increased cardiovascular disease (CVD) across all women with PMOS remains uncertain. Though the evidence for coronary heart disease and stroke remains inconclusive [4], several cohort studies and meta-analyses have reported increased risks of myocardial infarction, ischaemic heart disease, stroke and cardiovascular mortality [5,6,7,8,9], whereas others have found no increase in myocardial infarction or midlife cardiovascular risk [10,11]. This heterogeneity may, at least in part, reflect differences in obesity, insulin resistance and PMOS phenotype between study populations.

**Figure 1:**
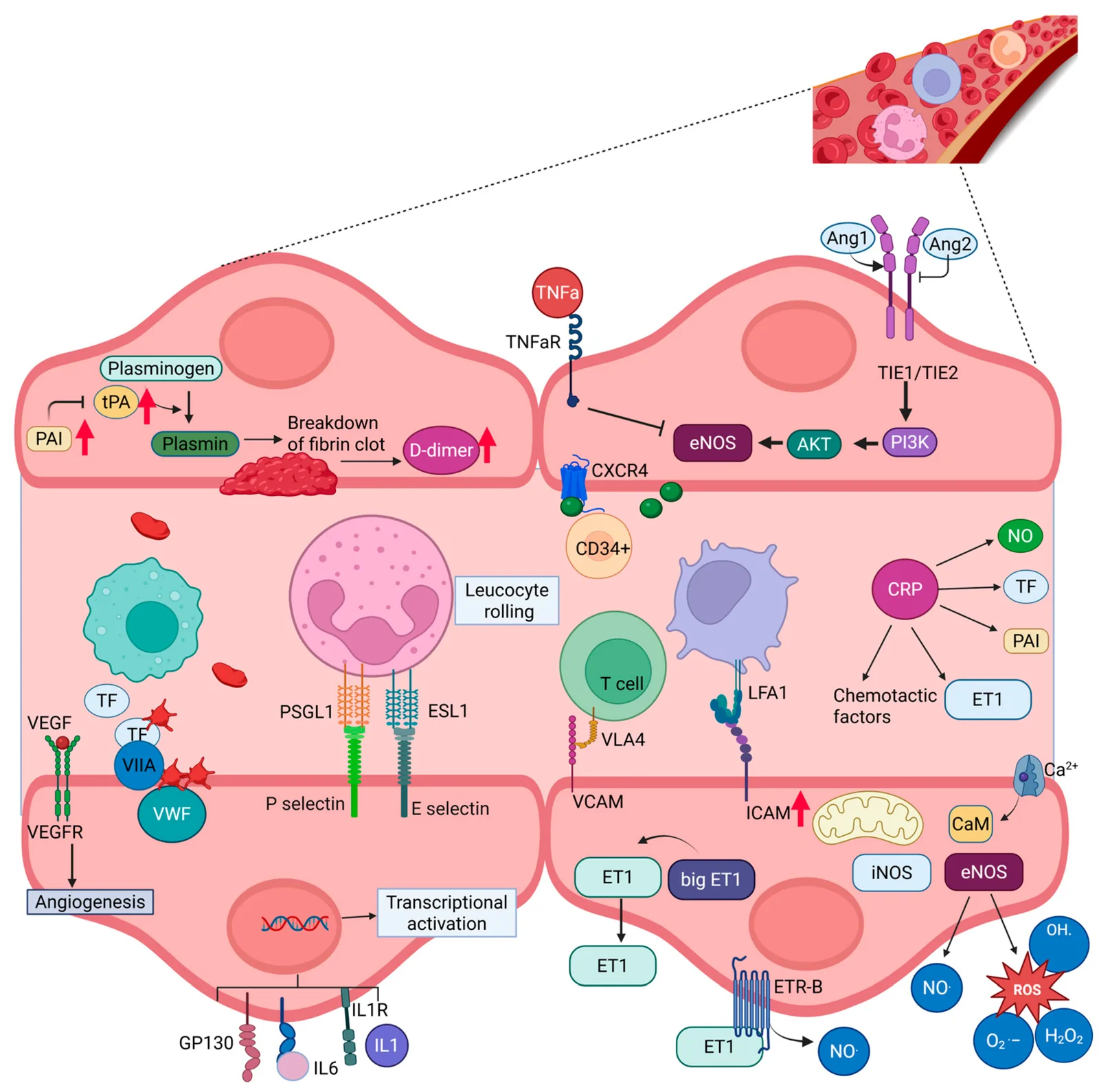
A schematic illustrating the differing groups of proteins associated with the development endothelial dysfunction. PAI: plasminogen activator inhibitor; tPA: tissue plasminogen activator; TNF-α: tumour necrosis factor-alpha; TNF-αR: tumour necrosis factor-alpha receptor; CXCR4: C-X-C motif chemokine receptor 4; eNOS: endothelial nitric oxide synthase; PI3K: phosphoinositide 3-kinase; AKT: protein kinase B; TIE: tyrosine kinase with Ig and EGF homology domains; CD34: cluster of differentiation 34; CRP: C-reactive protein; NO: nitric oxide; TF: tissue factor; ET1: endothelin-1; LFA1: lymphocyte function-associated antigen 1; VLA4: very late antigen-4 (also known as integrin α_4_β_1_ or CD49d/CD29); VCAM: vascular cell adhesion molecule; ICAM1: intercellular adhesion molecule-1; E-Selectin: endothelial-selectin; P-Selectin: platelet-selectin; CaM: calmodulin; iNOS: inducible nitric oxide synthase; PSGL1: P-selectin glycoprotein ligand-1; ESL1: E-selectin ligand-1; VEGF: vascular endothelial growth factor; VEGFR: vascular endothelial growth factor receptor; VWF: von Willebrand factor; GP130: glycoprotein 130; IL-6: interleukin-6; IL-1R: interleukin-1 receptor; IL-1: interleukin-1; ETR-B: endothelin receptor type-B; OH: hydroxyl radical; O_2_^−^: superoxide ion; H_2_O_2_: hydrogen peroxide; Ang1: angiotensin1; Ang2: angiotensin2; VII: factor VII (a blood clotting protein); T Cells: thymus-derived lymphocytes; Ca^2+^: calcium ion. Created in BioRender. Bahrain team 2, R. (2026) https://BioRender.com/58t8jb9.

Endothelial dysfunction represents an early stage in the development of vascular disease and may therefore provide insight into cardiovascular risk before clinically overt CVD becomes apparent [12]. Endothelial activation is associated with alterations in several classes of circulating proteins, including adhesion molecules such as intercellular adhesion molecule-1 (ICAM-1), vascular cell adhesion molecule-1 (VCAM-1) and E-selectin [13]; angiogenic and vascular-integrity proteins including angiopoietin-1, angiopoietin-2, Tie-2 and vascular endothelial growth factor (VEGF) [14]; and haemostatic proteins including P-selectin, D-dimer, plasminogen activator inhibitor-1 (PAI-1), tissue plasminogen activator (tPA) and von Willebrand factor (vWF) [15,16,17] (Figure 1). These circulating biomarkers provide complementary information concerning endothelial activation, vascular integrity, inflammation, coagulation and fibrinolysis, although they should not be considered equivalent to direct physiological measurements of endothelial function. Endothelial dysfunction additionally involves impaired nitric oxide bioavailability, oxidative stress, inflammatory signalling and altered endothelial repair. Interactions between endoplasmic-reticulum stress and the renin–angiotensin system have also been implicated in endothelial dysfunction through effects on nitric oxide availability, angiogenesis and oxidative-stress pathways [18].

Obesity is an important potential modifier of endothelial dysfunction in PMOS. In our recent study of women with PMOS and body mass index (BMI) > 30 kg/m^2^ who were BMI-matched to controls, circulating ICAM-1, tPA, PAI-1 and D-dimer concentrations were increased in PMOS [19]. These findings suggested a pattern of endothelial activation, impaired fibrinolysis and coagulation activation in obese PMOS. However, biochemical evidence of endothelial activation does not invariably correspond with abnormalities detected by physiological vascular measurements, and studies using reactive hyperaemia peripheral arterial tonometry, brachial artery responses and flow-mediated dilatation have produced inconsistent findings in PMOS [20,21,22,23,24]. Importantly, obesity and its associated metabolic abnormalities are potential confounders in many of these studies. Whether a comparable circulating endothelial phenotype is present in non-obese women with PMOS therefore remains unclear.

Vitamin D represents another potential modifier of the vascular phenotype in PMOS. Vitamin D_3_ is converted to 25-hydroxyvitamin D_3_ [25(OH)D_3_], while subsequent metabolism produces biologically active 1,25-dihydroxyvitamin D_3_ [1,25(OH)_2_D_3_] and the catabolic metabolite 24,25-dihydroxyvitamin D_3_ [24,25(OH)_2_D_3_] [25]. Lower vitamin D concentrations have been associated with obesity, insulin resistance and hyperandrogenism, all of which are common features of PMOS [26,27]. Vitamin D signalling has also been implicated in inflammatory, oxidative-stress and mitochondrial pathways that may influence endothelial biology [28]. Experimental evidence also supports a potential direct effect of active vitamin D signalling on endothelial inflammatory responses; 1,25(OH)_2_D_3_ has recently been reported to attenuate TNF-α-induced endothelial cell injury through modulation of TNF-α/NF-κB signalling [29]. However, evidence for an independent anti-inflammatory or vasculoprotective effect of circulating 25(OH)D_3_ remains inconsistent [30], although vitamin D supplementation may modify selected inflammatory markers [31], and the immunomodulatory actions of 1,25(OH)_2_D_3_ are better established [32,33,34]. Consequently, associations between vitamin D status and endothelial dysfunction in PMOS may be confounded by adiposity and metabolic dysfunction.

Obesity is strongly associated with endothelial dysfunction and represents an important potential confounder when evaluating vascular abnormalities in PMOS. Studying non-obese women therefore provides an opportunity to reduce the dominant influence of adiposity and to examine whether PMOS itself, or its associated endocrine and metabolic abnormalities, is related to endothelial activation. The present exploratory study compared a broad panel of circulating endothelial, inflammatory, coagulation and fibrinolytic biomarkers in age- and BMI-matched non-obese women with and without PMOS and examined their relationships with insulin resistance, hyperandrogenism, AMH and vitamin D metabolites.

We hypothesised that, in the absence of obesity and major between-group differences in insulin resistance, systemic inflammation and vitamin D status, circulating biomarkers of endothelial dysfunction would not differ significantly between women with PMOS and controls. We additionally explored whether inter-individual variation in insulin resistance, androgen excess, AMH and vitamin D metabolites was associated with specific endothelial biomarkers.

## 2. Results

Fifty-eight women were included in the study, comprising 29 women with PMOS and 29 healthy controls. The demographic, endocrine and biochemical characteristics of the study population are presented in Table 1. As expected, women with PMOS demonstrated significantly higher FAI, Ferriman–Gallwey scores and AMH concentrations compared with controls, confirming the expected hyperandrogenic phenotype. BMI, age and other routine biochemical variables were broadly comparable between groups. Serum CRP concentrations did not differ significantly between women with PMOS and controls.

### 2.1. Vitamin D Metabolites

Serum concentrations of 1,25(OH)_2_D_3_, 25(OH)D_3_, 25(OH)D_2_ and 24,25(OH)_2_D_3_ did not differ significantly between women with PMOS and controls (Table 1), indicating that overall vitamin D status was similar in both groups.

### 2.2. Inflammatory and Endothelial Biomarkers

Comparison of endothelial biomarkers between women with PMOS and controls demonstrated no differences after correction for multiple testing (Table 2). Although several markers demonstrated moderate effect sizes, including ICAM-5, VEGF-A, angiopoietin-1 and ICAM-2, none remained significant following Benjamini–Hochberg FDR correction, suggesting that systemic inflammatory and endothelial profiles were broadly comparable between groups.

Values are mean ± SD and are reported as relative fluorescence units (RFU). Between-group comparisons were performed using two-sided Welch’s independent-samples *t*-tests. *p*-values were adjusted for multiple comparisons using the Benjamini–Hochberg false discovery rate (BH-FDR) procedure. Positive effect sizes indicate higher concentrations in PMOS and negative values indicate lower concentrations in PMOS. Hedges’ g represents Cohen’s d corrected for small-sample bias; 95% confidence intervals (CI) indicate the precision of the effect-size estimate. No between-group comparison remained statistically significant following FDR correction.

### 2.3. Correlations Between Vitamin D Metabolites and Inflammatory Markers

Correlation analyses evaluating the relationships between vitamin D metabolites and inflammatory/endothelial biomarkers showed no associations following FDR correction, for the combined cohort or control group (Supplementary Tables S1–S3).

Within the PMOS cohort, a significant positive association was observed between 24,25(OH)_2_D_3_ and TNF (Pearson *r* = 0.648, *p* = 0.002, FDR-adjusted *q* = 0.042). Although 25(OH)D_3_ demonstrated a positive correlation with TNF (*r* = 0.561, *p* = 0.010), this association did not remain significant after correction for multiple testing. No other vitamin D metabolite demonstrated robust associations with inflammatory or endothelial biomarkers.

### 2.4. Associations of HOMA-IR, FAI and AMH with Vitamin D Metabolites and Inflammatory Biomarkers

Correlation analyses were performed between HOMA-IR, FAI, AMH, vitamin D metabolites and inflammatory/endothelial biomarkers in the combined cohort and separately within the PMOS and control groups (Table 3).

In the combined cohort, insulin resistance was positively associated with P-selectin (Pearson *r* = 0.502, FDR-adjusted *q* = 0.021) and ICAM-2 (*r* = 0.436, FDR-adjusted *q* = 0.049). FAI showed a positive association with E-selectin (*r* = 0.439, FDR-adjusted *q* = 0.049).

Within women with PMOS, FAI demonstrated a positive association with E-selectin (Pearson *r* = 0.718, *p* < 0.001), with the analysis remaining significant following FDR correction (Pearson *q* = 0.027). HOMA-IR was also positively associated with ICAM-2 (Pearson *r* = 0.670, *p* = 0.001; FDR-adjusted *q* = 0.046).

Among control women, HOMA-IR demonstrated a positive correlation with P-selectin (Pearson *r* = 0.738, *p* < 0.001; FDR-adjusted *q* = 0.001), representing the strongest association identified in the study.

There were no associations between HOMA-IR, FAI or AMH and any vitamin D metabolite after FDR correction. Several nominal associations were identified, including positive correlations between FAI and 1,25(OH)_2_D_3_ and inverse correlations between AMH and both 1,25(OH)_2_D_3_ and tissue-type plasminogen activator (tPA) (Supplementary Tables S4–S6).

Because several endothelial biomarkers demonstrated departures from normality, Spearman rank correlations were performed as sensitivity analyses, with Benjamini–Hochberg FDR correction applied using the same multiple-testing framework as the primary analyses (Table 4). The association between FAI and E-selectin in women with PMOS was highly robust (Spearman ρ = 0.720, *p* < 0.001, FDR-adjusted *q* = 0.009), closely reproducing the primary Pearson result (*r* = 0.718, FDR-adjusted *q* = 0.027). The association between HOMA-IR and ICAM-2 in PMOS remained positive and nominally significant using Spearman analysis (ρ = 0.579, *p* = 0.007) but did not remain significant following FDR correction (*q* = 0.129). Similarly, the HOMA-IR–P-selectin association in controls remained nominally significant (ρ = 0.473, *p* = 0.011) but not after FDR correction (*q* = 0.273). The positive association between 24,25(OH)_2_D_3_ and TNF in PMOS was also attenuated in the sensitivity analysis (ρ = 0.519, *p* = 0.019, *q* = 0.361). Of note, FAI was also positively associated with 1,25(OH)_2_D_3_ in women with PMOS in the Spearman sensitivity analysis (ρ = 0.708, *p* = 0.001, *q* = 0.018). As this association was not significant in the primary FDR-adjusted Pearson analysis, it should be regarded as exploratory. 

## 3. Discussion

**Figure 2:**
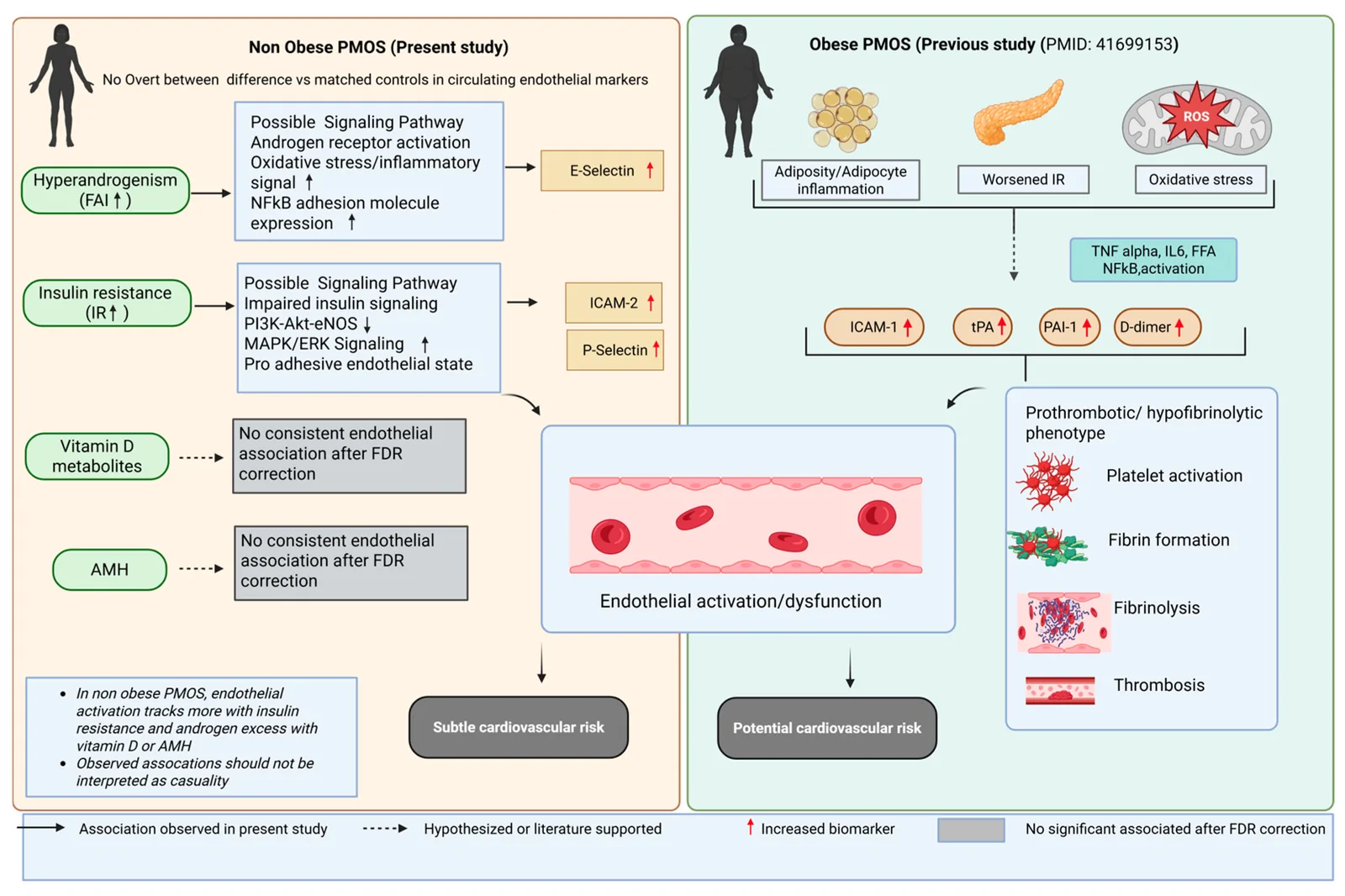
Schematic summary of the principal associations identified in the present study and their relationship to previous findings in obese PMOS [19]. Solid arrows indicate statistically significant associations after Benjamini–Hochberg FDR correction in the present dataset and should not be interpreted as evidence of causality. Dashed connections indicate hypotheses- or literature-based interpretation. In non-obese women with PMOS, HOMA-IR was positively associated with ICAM-2 and FAI with E-selectin, whereas vitamin D metabolites and AMH showed no consistent associations with endothelial biomarkers after FDR correction. Previous findings in obese PMOS suggest a broader prothrombotic/hypofibrinolytic biomarker profile. FFAs: free fatty acids; ROS: reactive oxygen species; FAI: free androgen index; IR: insulin resistance; Enos: endothelial nitric oxide synthase; TNF-α: tumour necrosis factor-alpha; IL-6: interleukin-6; AMPK: AMP-activated protein kinase; BH4: tetrahydrobiopterin; PI3K: phosphoinositide 3-kinase; Akt: protein kinase B; NF-κB: nuclear factor kappa-light-chain-enhancer of activated B cells; MAPKs: mitogen-activated protein kinase; PI3K: phosphoinositide 3-kinase; ERK: extracellular signal-regulated kinase; ICAM-1: intercellular adhesion molecule-1; ICAM-2: intercellular adhesion molecule-2; E-Selectin: endothelial-Selectin; P-selectin: platelet selectin; PAI-1: plasminogen activator inhibitor-1; tPA: tissue plasminogen activator; AMH: anti-Müllerian hormone; FDR: false discovery rate; NF-Κb: nuclear factor kappa-light-chain-enhancer of activated B cells; PMOS: polyendocrine metabolic ovarian syndrome. The illustration was created in BioRender Nandakumar, M. (2026) https://BioRender.com/fyxu3j8 (with publication licence Date: 20 August 2026).

This study evaluated whether vitamin D metabolites are associated with endothelial dysfunction and inflammatory biomarkers in women with PMOS. We hypothesised that, in weight-matched women who were not obese, endothelial cell dysfunction protein markers would not differ between those with and without PMOS and would be independent of vitamin D levels. In accord with our original hypothesis, circulating concentrations of 1,25(OH)_2_D_3_, 25(OH)D_3_, 25(OH)D_2_ and 24,25(OH)_2_D_3_ were not significantly different between women with PMOS and controls [35], nor were they significantly associated with endothelial dysfunction after correction for multiple testing. The most robust finding was the positive association between FAI and E-selectin in women with PMOS, which remained essentially unchanged in magnitude and statistically significant following both Pearson and Spearman analyses with FDR correction (Figure 2). The association between HOMA-IR and ICAM-2 was significant in the primary Pearson analysis and remained positive and nominally significant in the Spearman sensitivity analysis, although it no longer survived FDR correction. These findings provide stronger evidence for an association between hyperandrogenism and endothelial activation than for insulin resistance within this relatively small non-obese cohort, and that this suggests that endothelial dysfunction in non-obese PMOS is more closely associated with the metabolic and endocrine abnormalities characteristic of PMOS than with circulating vitamin D status.

In our previous investigation of BMI-matched obese women with PMOS, women with PMOS demonstrated significantly higher circulating ICAM-1, tissue plasminogen activator (tPA), plasminogen activator inhibitor-1 (PAI-1) and D-dimer concentrations than BMI-matched controls, consistent with a broader pattern of endothelial activation, altered fibrinolysis and coagulation activation [19]. This contrasts with the present non-obese cohort, in which no overall between-group differences in endothelial biomarkers remained significant after correction for multiple testing. Instead, variation in selected endothelial activation markers was associated with HOMA-IR and FAI. Differences between the studies may reflect not only adiposity but also PMOS phenotype and associated metabolic severity. The obese cohort predominantly represented phenotype A, whereas the present cohort represented phenotype B, which may be associated with a less adverse metabolic profile. Taken together, these observations raise the possibility that the circulating endothelial phenotype of PMOS varies according to adiposity and metabolic/phenotypic characteristics. Obese PMOS was associated with a broader prothrombotic and hypofibrinolytic biomarker profile characterised by increased ICAM-1, PAI-1, tPA and D-dimer [18], whereas in the present non-obese cohort, endothelial biomarker variation was more closely associated with insulin resistance and androgen excess (Figure 2). This interpretation is consistent with previous evidence demonstrating that obesity amplifies cardiovascular risk in PMOS [9] while insulin resistance remains an intrinsic feature of the syndrome even in lean women [36]. These cross-sectional comparisons cannot establish a progression between phenotypes, but they support further investigation of whether obesity and worsening metabolic dysfunction amplify vascular abnormalities in PMOS.

Women with PMOS have an increased risk of cardiovascular disease, with recent meta-analyses demonstrating increased risks of myocardial infarction, stroke and other cardiovascular events [5,6,7,8,11,12,15,16]. Endothelial dysfunction is believed to represent one of the earliest manifestations of this increased cardiovascular risk, preceding clinically overt vascular disease [28]. Experimental and clinical studies have demonstrated impaired endothelial-dependent vasodilatation in women with PMOS, although findings have been inconsistent owing to differences in obesity, insulin resistance and study design [20,21,22,23,24].

The observed associations are biologically plausible. Insulin resistance is accompanied by abnormalities in endothelial insulin signalling, reduced nitric oxide bioavailability and enhanced pro-inflammatory and pro-adhesive signalling, providing potential pathways through which increasing metabolic dysfunction may accompany endothelial activation. P-selectin participates in platelet–leukocyte interactions and vascular inflammation, whereas ICAM-2 regulates leukocyte adhesion and transendothelial migration [37,38]. Hyperandrogenism may provide an additional vascular influence. Our findings are consistent with previous mechanistic studies demonstrating adverse vascular effects of androgen excess. Previous mechanistic studies have implicated androgen-dependent endothelin receptor signalling and altered endothelial nitric oxide production in women with PMOS [39]. Excess androgens may impair microvascular endothelial function through endothelin receptor signalling [40], while others have shown that androgen excess suppresses endothelial nitric oxide production and that this dysfunction can be reversed by estradiol administration [39]. Similarly, it was reported that endothelial dysfunction in young women with PMOS was associated with insulin resistance and low-grade inflammation [41]. E-selectin is induced during endothelial activation and therefore provides a plausible marker through which an association with androgen excess may be detected [42]. Although these mechanisms provide biological context for the observed correlations, the present cross-sectional data cannot determine whether insulin resistance or hyperandrogenism directly caused the endothelial biomarker changes. Taken together with previous mechanistic studies, these observations support an association between metabolic dysfunction, hyperandrogenism and endothelial activation in PMOS, while the direction and causal nature of these relationships require confirmation in prospective or interventional studies.

Although no between-group endothelial biomarker difference remained statistically significant after FDR correction, several markers demonstrated moderate effect-size estimates, including ICAM-5, VEGF-A, angiopoietin-1 and ICAM-2. These observations should not be interpreted as evidence of established between-group differences, particularly given the accompanying wide confidence intervals. Nevertheless, the magnitude and direction of these effects may be useful for hypothesis generation and for informing sample-size calculations in larger independent confirmatory cohorts.

AMH was included as an exploratory marker of the ovarian/reproductive phenotype of PMOS. Although AMH concentrations were substantially higher in women with PMOS, AMH was not significantly associated with vitamin D metabolites or inflammatory/endothelial biomarkers after FDR correction. This contrasts with the significant associations observed for HOMA-IR and FAI and suggests that, within this cohort, variation in selected endothelial biomarkers was more closely associated with metabolic and androgenic features than with AMH as a marker of ovarian follicular activity. Nevertheless, the modest sample size limits our ability to exclude weaker relationships involving AMH. The absence of robust endothelial associations with AMH should also be viewed within the broader phenotypic heterogeneity of PMOS. AMH is an important marker of the reproductive/follicular component of the syndrome and has been associated with reproductive prognosis in women with PMOS undergoing assisted reproduction [43]. The present findings suggest that the determinants of reproductive phenotype and those associated with circulating endothelial activation need not be identical: in this cohort, HOMA-IR and FAI, rather than AMH, showed the more robust associations with selected endothelial biomarkers. This supports the consideration of metabolic, androgenic and reproductive aspects as related, but distinct components of PMOS heterogeneity.

Clinically, these findings emphasise that absence of obesity should not be assumed to imply absence of potentially adverse vascular associations in PMOS. Assessment of conventional metabolic risk factors, including insulin resistance and androgen excess, may remain relevant in non-obese women. However, the present exploratory data do not establish P-selectin, ICAM-2 or E-selectin as clinical screening biomarkers, and prospective studies linking these proteins to validated functional vascular measures or cardiovascular outcomes are required before they can be considered for individual risk stratification.

Circulating vitamin D metabolites showed few associations with endothelial dysfunction. Although several nominal correlations were identified, none remained statistically significant following FDR correction. These findings suggest that vitamin D deficiency is unlikely to represent a major independent determinant of endothelial dysfunction within this cohort [44]. This is consistent with recent evidence indicating that many reported associations between vitamin D deficiency and cardiovascular risk in PMOS are attenuated after adjustment for adiposity and metabolic factors. It is therefore possible that, clinically, vitamin D deficiency is primarily a marker of adverse metabolic health rather than a direct mediator of endothelial injury [45]. The concentrations of 1,25(OH)_2_D_3_, 25(OH)D_3_, 25(OH)D_2_ and 24,25(OH)_2_D_3_ were similar between women with PMOS and controls, reducing the likelihood that between-group differences in vitamin D status could account for the endothelial findings. Interpretation should nevertheless consider that vitamin D status is influenced by season, ultraviolet-light exposure, dietary intake and supplementation. These exposures were not comprehensively characterised in the present study, and residual variability related to them cannot be excluded. The relatively narrow metabolic differences between these non-obese, BMI-matched cohorts may also have reduced the ability to detect relationships reported in populations with more pronounced adiposity or metabolic dysfunction. Prospective studies incorporating seasonal sampling, detailed assessment of dietary vitamin D and supplementation, and, where appropriate, intervention to correct vitamin D deficiency would be required to determine whether modification of vitamin D status has a measurable effect on endothelial biomarkers or functional vascular outcomes in women with PMOS.

No significant differences were observed in circulating TNF, IL-1α or IL-6 concentrations between groups. These findings suggest that endothelial activation may occur independently of overt systemic inflammation or reflect predominantly local vascular inflammation that is not adequately captured by circulating cytokine measurements. Similar dissociation between endothelial activation markers and inflammatory cytokines has been described in other cardiometabolic disorders [23,25]. However, circulating cytokine concentrations provide only a systemic measure of inflammation and may not reflect tissue-resident, endothelial or vascular-wall inflammatory activity. Consequently, the absence of differences in circulating cytokines should not be interpreted as evidence for the absence of local vascular inflammation. The use of a highly multiplexed aptamer-based proteomic platform allowed simultaneous assessment of a broad endothelial biomarker panel; however, assay-specific analytical characteristics may influence the detection of small between-group differences. SOMAscan measurements represent relative protein abundance, and subtle effects should ideally be replicated using orthogonal targeted assays in an independent cohort. Accordingly, absence of a statistically significant difference in the present study should not be interpreted as proof of biological equivalence.

The present study has several strengths. The study addresses a gap in the literature by comparing endothelial dysfunction markers in BMI-matched non-obese women with and without PMOS. This allowed comparison of endothelial dysfunction markers and vitamin D metabolites and their correlation without the bias of BMI. Furthermore, the use of both Pearson and Spearman correlation analyses with Benjamini–Hochberg false discovery rate correction substantially reduced the likelihood of false-positive findings arising from multiple comparisons. Several limitations should also be acknowledged. First, the modest sample size limits statistical power, particularly following correction for multiple comparisons, and increases uncertainty around effect-size estimates. The study should therefore be regarded as exploratory and hypothesis-generating. Second, its cross-sectional design precludes conclusions regarding the direction or causality of the observed associations between HOMA-IR, FAI and endothelial biomarkers. Third, circulating protein concentrations represent surrogate biomarkers and do not provide a direct physiological measurement of endothelial function; concurrent assessment using methods such as flow-mediated dilatation or peripheral arterial tonometry would strengthen future studies. Fourth, measurement of circulating inflammatory proteins cannot fully characterise tissue-resident or vascular-wall inflammatory signalling; local endothelial inflammation could therefore be present despite similar systemic cytokine concentrations. Fifth, seasonal variation in vitamin D exposure, dietary intake and supplementation was not comprehensively characterised. Finally, the highly multiplexed SOMAscan platform provides relative protein measurements, and subtle findings should be confirmed using orthogonal assays and larger independent cohorts. Future studies should incorporate longitudinal assessment to determine whether changes in insulin resistance or androgen exposure precede changes in endothelial activation. Interventional studies examining whether improvement in insulin sensitivity, modification of androgen excess or correction of vitamin D deficiency alters endothelial biomarkers and direct measures of vascular function would be particularly informative.

## 4. Materials and Methods

### 4.1. Study Design

In this exploratory cross-sectional study, plasma endothelial-associated protein levels were determined in women with PMOS (*n* = 29) and control (*n* = 29) women, all recruited from Hull IVF clinic [25]. Control and PMOS women were age- and BMI-matched. All procedures were conducted in accordance with the ethical standards of the Yorkshire and The Humber NRES ethical committee, UK (ethics number 02/03/043), which granted approval for the study. For PMOS diagnosis, Rotterdam consensus criteria were employed: (1) biochemical (free androgen index (FAI) > 4) and clinical (Ferriman–Gallwey score > 8) hyperandrogenaemia; (2) amenorrhea or oligomenorrhea; and (3) transvaginal ultrasound diagnosis of polycystic ovaries [46]. No other condition/illness was present in the PMOS study participants, and all were medication-free (including no over-the-counter medications) for ≥9 months prior to enrolment. Testing to exclude the following endocrine conditions was undertaken: Cushing’s disease, hyperprolactinemia, non-classical 21-hydroxylase deficiency, or an androgen-secreting tumour [47]. Control participants were recruited from the same clinical setting as women with PMOS and underwent clinical and biochemical assessment. Participants with known coexisting illness were excluded, and all participants were medication-free, including over-the-counter medication, for at least nine months before enrolment. Testing was undertaken to exclude endocrine disorders that could mimic features of PMOS, including Cushing’s disease, hyperprolactinaemia, non-classical 21-hydroxylase deficiency and an androgen-secreting tumour [47]. Controls did not fulfil diagnostic criteria for PMOS. These criteria were intended to minimise the influence of recognised endocrine or systemic disease on the measured biomarkers.

Blood sampling was performed on day 21 of the menstrual cycle in regularly cycling participants; the timing protocol for women with oligomenorrhoea/amenorrhoea was performed on day 21 following a progesterone-induced bleed. None of the participants was receiving hormonal therapy, and all had been medication-free for at least nine months before enrolment. After overnight fasting, waist circumference, height (centimetres) and weight (kilogrammes) (to calculate body mass index (BMI) using the formula kg/m^2^) were measured per WHO guidelines [48]. Fasting bloods were centrifuged (3500× *g* 15 min), aliquoted and stored (−80 °C). Anti-Müllerian hormone (AMG), Sex hormone binding globulin (SHBG), insulin (DPC Immulite 200 analyser, Euro/DPC, Llanberis, UK), and plasma glucose (for calculation of homeostasis model assessment-IR (HOMA-IR)) (Synchron LX20 analyser, Beckman-Coulter, High Wycombe, UK) were measured. FAI was calculated by dividing total testosterone by SHBG × 100. Serum testosterone and vitamin D were determined by isotope-dilution liquid chromatography tandem mass spectrometry (LC-MS/MS: Waters corporation, Wilmslow, UK) [25,49].

SOMAscan proteomic analysis: Plasma proteins were quantified using the SOMAscan V3.2 HTS platform (SomaLogic, Boulder, CO, USA), using SOMAscan menu version 1.3k_v01. Protein abundance was reported as relative fluorescence units (RFUs). According to the original assay metadata, raw RFU data underwent sequential intraplate hybridisation normalisation, plate scaling, intraplate median normalisation stratified by sample type, calibration and filtering. The plate-scaling and calibration reference was EDTAPlasma_Tec_Cal_SL16485_8p_1.3k_mm3.4_p3.2. Plate-scale scalar values were 1.601 for Set 002 (PASS) and 2.877 for Set 001 (FLAG). Plate median calibration factors were 1.007 and 1.000 for Sets 002 and 001, respectively, with both passing the plate-median test. Plate-tail percentages were 10.0% for Set 002 and 6.1% for Set 001 and were flagged in the original assay metadata. The assay incorporated 0.005%, 1% and 40% dilution groups. Filtering was performed using SOMAscan menu version 1.3k_v01, with scaleFactor specified as the assay QC pass/fail criterion. The original output reports PASS/FLAG classifications but does not specify a numerical threshold for the scaleFactor criterion. Following the manufacturer’s processing pipeline, normalised RFU values for the prespecified endothelial-associated proteins were extracted for the present analysis; no additional investigator-defined filtering thresholds were applied. The SOMAscan Assay was used to specifically target those endothelial biomarker proteins in the SOMAscan panel. Markers of endothelial activation/dysfunction: these included vascular endothelial growth factor (VEGF), E-selectin, intercellular adhesion molecule (ICAM)-1,2,3,5, vascular cell adhesion molecule-1 (VCAM-1), P-selectin and cadherin-5. Markers of vascular integrity: angiopoietin-1 (Ang-1) and angiopoietin-2 (Ang-2), Tie-2 receptor and von Willebrand Factor (vWF). Inflammatory markers; C-reactive protein (CRP); tumour necrosis factor-alpha (TNF-α); pro-inflammatory cytokines, interleukins (IL-1, IL-6). Markers of coagulation and fibrinolysis: tissue factor (TF), tissue plasminogen activator (tPA), plasminogen activator inhibitor-1 (PAI-1) and D-dimer.

### 4.2. Statistics

This was an exploratory study. Because no previous study had evaluated this endothelial biomarker panel in a comparable non-obese PMOS population or examined its relationships with vitamin D metabolites, a reliable effect size on which to base an a priori formal power calculation was unavailable. The sample size was therefore determined by the availability of well-characterised age- and BMI-matched samples, and the analyses should be regarded as hypothesis-generating. Effect sizes and their 95% confidence intervals are reported to facilitate interpretation of the magnitude and precision of the observed differences and to inform future confirmatory studies.

We assessed continuous-variable distributions using the Shapiro–Wilk test and found that several endothelial biomarkers departed from normality, whereas the principal vitamin D metabolites were approximately normally distributed. Given the balanced group design, Welch’s *t*-test was retained for between-group comparisons. Welch’s test was selected because it does not require equality of group variances. Mean between-group differences and 95% confidence intervals (CIs) were calculated using the Welch–Satterthwaite approximation for degrees of freedom. Effect sizes are reported as Hedges’ g, which provides a small-sample correction to Cohen’s d. Between-group effect estimates are presented as mean differences with 95% CIs and Hedges’ g with 95% CIs. Given the modest sample size and the age- and BMI-matched design, additional covariate-adjusted models were not undertaken in order to avoid overfitting exploratory analyses.

For Pearson correlation analyses, scatterplots were inspected to assess approximate linearity and to identify potentially influential observations; because several biomarker distributions departed from normality, Spearman rank correlations were additionally performed as sensitivity analyses (Table 4).

To account for multiple testing, *p*-values within the predefined families of comparisons were adjusted using the Benjamini–Hochberg false discovery rate (FDR) procedure. FDR-adjusted *q*-values < 0.05 were considered statistically significant. Because of the exploratory nature of the study, both unadjusted *p*-values and FDR-adjusted *q*-values are reported, together with effect sizes and 95% CIs where appropriate. All statistical tests were two-sided.

There were no missing data for the variables included in the reported analyses; correlation analyses were specified to use pairwise complete observations. Analyses were performed using R version 4.0.0 (R Foundation for Statistical Computing, Vienna, Austria).

## 5. Conclusions

In conclusion, non-obese women with PMOS did not demonstrate a generalised alteration in circulating biomarkers of endothelial dysfunction compared with age- and BMI-matched controls. Within PMOS, however, insulin resistance and hyperandrogenism were associated with selected markers of endothelial activation, whereas vitamin D metabolites showed little consistent relationship with the endothelial biomarker profile. Comparison with our previous findings in obese PMOS raises the possibility that adiposity and metabolic phenotype modify the vascular biomarker profile of PMOS, with broader prothrombotic and hypofibrinolytic abnormalities occurring in metabolically more adverse phenotypes. These cross-sectional observations are hypothesis-generating and require confirmation in larger longitudinal studies incorporating direct functional measures of endothelial function.

## Figures and Tables

**Table 1:** Clinical and biochemical characteristics of the women with polyendocrine metabolic ovary syndrome (PMOS) and control women. Data is presented as mean ± standard deviation (SD). BMI, body mass index; TSH, thyroid stimulating hormone; FAI, free androgen index; AMH, anti-Mullerian hormone; FG score, Ferriman–Gallwey score; CRP, C-reactive protein.

Characteristic	PMOS Mean ± SD	Controls Mean ± SD	Mean Difference (95% CI)	Welch t (df)	*p*-Value	Hedges’ g
Age (years)	31.6 ± 5.3	32.9 ± 4.4	−1.36 (−4.31 to 1.58)	−0.94 (36.4)	0.35	−0.27
BMI (kg/m^2^)	25.5 ± 3.7	25.3 ± 3.6	0.24 (−1.97 to 2.45)	0.22 (40.3)	0.82	0.06
Fasting glucose (mmol/L)	4.5 ± 0.3	4.8 ± 0.3	−0.25 (−0.46 to −0.04)	−2.43 (41.1)	0.019	−0.69
Insulin (μIU/L)	7.3 ± 2.7	7.6 ± 4.1	−0.32 (−2.32 to 1.68)	−0.33 (45.8)	0.74	−0.08
HOMA-IR	1.50 ± 0.6	1.68 ± 1.0	−0.19 (−0.67 to 0.29)	−0.79 (45.0)	0.43	−0.2
TSH (mIU/L)	2.96 ± 3.4	2.03 ± 0.7	0.93 (−0.72 to 2.58)	1.17 (20.3)	0.25	0.39
17 alpha progesterone (nmol/L)	7.49 ± 4.4	6.24 ± 5.6	1.25 (−1.90 to 4.41)	0.80 (39.3)	0.42	0.24
Prolactin (mIU/L)	352 ± 126	290 ± 124	62.08 (−14.83 to 139.00)	1.63 (40.4)	0.11	0.48
FAI	3.5 ± 2.4	1.2 ± 0.4	2.27 (1.10 to 3.43)	4.05 (19.9)	0.0006	1.37
AMH (ng/mL)	53.2 ± 15.4	23.5 ± 13.7	29.71 (20.99 to 38.44)	6.89 (38.0)	3.41862 × 10^−8^	2.024
FG score	12.2 ± 2.4	3.2 ± 1.3	9.04 (7.80 to 10.27)	15.04 (26.8)	1.3483 × 10^−14^	4.75
CRP (mg/L)	2.5 ± 2.1	2.5 ± 2.3	−0.03 (−1.37 to 1.31)	−0.04 (42.8)	0.96	−0.012
1,25(OH)_2_D_3_ (ng/mL)	0.05 ± 0.02	0.05 ± 0.02	0.00 (−0.01 to 0.01)	0.28 (34.8)	0.78	0.089
25(OH)D_3_ (ng/mL)	23.2 ± 10.9	22.7 ± 11.5	0.46 (−6.14 to 7.06)	0.14 (42.4)	0.89	0.03
25(OH)D_2_ (ng/mL)	0.59 ± 0.24	0.83 ± 0.48	−0.23 (−0.48 to 0.01)	−1.96 (34.9)	0.06	−0.54
24,25(OH)_2_D_3_ (ng/mL)	1.08 ± 0.68	1.02 ± 0.65	0.07 (−0.33 to 0.46)	0.34 (39.8)	0.73	0.091

**Table 2:** Comparison of inflammatory and endothelial markers between women with PMOS and controls.

Marker	PMOS (*n* = 29), Mean ± SD (RFU)	Controls (*n* = 29), Mean ± SD (RFU)	Welch’s t (df)	*p*-Value	BH-FDR *q* Value	Hedges’ g (95% CI)
E-selectin	21,109 ± 9888	20,825 ± 8458	0.10 (36.9)	0.918	0.963	0.03 (−0.53 to 0.60)
ICAM-1	3014 ± 1338	3198 ± 1109	−0.50 (36.1)	0.618	0.898	−0.15 (−0.71 to 0.42)
VCAM-1	9698 ± 2919	9896 ± 2710	−0.24 (39.2)	0.813	0.898	−0.07 (−0.63 to 0.50)
Cadherin-5	10,909 ± 2165	11,474 ± 2218	−0.88 (41.7)	0.383	0.898	−0.25 (−0.82 to 0.32)
P-selectin	15,483 ± 11,097	15,360 ± 7714	0.04 (31.7)	0.966	0.966	0.01 (−0.55 to 0.58)
vWF	10,382 ± 10,393	11,421 ± 14,480	−0.29 (46.0)	0.774	0.898	−0.08 (−0.64 to 0.49)
ICAM-3	491.4 ± 127.6	541 ± 330	−0.74 (37.2)	0.466	0.898	−0.19 (−0.75 to 0.38)
ICAM-5	498 ± 108	556 ± 142	−1.59 (45.7)	0.119	0.898	−0.44 (−1.01 to 0.14)
ICAM-2	1432 ± 242	1680 ± 864	−1.44 (32.7)	0.161	0.898	−0.36 (−0.92 to 0.21)
VEGF-A	9405 ± 1425	8526 ± 2612	1.50 (43.5)	0.142	0.898	0.39 (−0.18 to 0.96)
Angiopoietin-1	545 ± 295	689 ± 429	−1.38 (46.0)	0.175	0.898	−0.37 (−0.94 to 0.20)
Angiopoietin-2	90.8 ± 24.3	93.4 ± 36.0	−0.30 (45.9)	0.765	0.898	−0.08 (−0.65 to 0.48)
Soluble Tie-2	1565 ± 401	1477 ± 418	0.74 (42.1)	0.461	0.898	0.21 (−0.35 to 0.78)
TNF	335 ± 49	343 ± 90	−0.39 (43.6)	0.698	0.898	−0.10 (−0.67 to 0.46)
IL-1α	591 ± 200	638 ± 284	−0.67 (46.0)	0.507	0.898	−0.18 (−0.75 to 0.38)
IL-6	293 ± 134	271 ± 69	0.68 (26.2)	0.502	0.898	0.22 (−0.35 to 0.78)
Tissue factor	917 ± 214	966 ± 423	−0.52 (42.1)	0.603	0.898	−0.14 (−0.70 to 0.43)
tPA	332 ± 179	318 ± 198	0.25 (43.4)	0.803	0.898	0.07 (−0.49 to 0.64)
PAI-1	241 ± 197	264 ± 190	−0.41 (40.1)	0.684	0.898	−0.12 (−0.68 to 0.45)
D-dimer	14,254 ± 4559	15,782 ± 10,543	−0.68 (39.2)	0.499	0.898	−0.17 (−0.74 to 0.39)
SDF-1	4079 ± 916	4256 ± 1034	−0.62 (43.8)	0.537	0.898	−0.18 (−0.74 to 0.39)

**Table 3:** Pearson correlation analysis (and false discovery rate: Pearson *q*) of HOMA-IR, FAI and AMH versus vitamin D and inflammatory/endothelial markers.

Cohort	Predictor	Outcome	Pearson *r*	Pearson *p*	Pearson *q*
Combined	HOMA-IR	P-selectin	0.502	0.000	0.021
Combined	FAI	E-selectin	0.439	0.002	0.049
Combined	HOMA-IR	ICAM-2	0.436	0.002	0.049
Controls	HOMA-IR	P-selectin	0.738	<0.0001	0.001
PMOS	FAI	E-selectin	0.718	<0.0001	0.027
PMOS	HOMA-IR	ICAM-2	0.670	0.001	0.046

**Table 4:** Spearman correlation analysis (and false discovery rate: Spearman *q*) of HOMA-IR, FAI and AMH versus vitamin D and inflammatory/endothelial markers. Values are Spearman rank correlation coefficients (ρ). *p*-values were adjusted for multiple comparisons using the Benjamini–Hochberg false discovery rate (FDR) procedure. Only associations with FDR-adjusted *q* < 0.05 are shown.

Cohort	Predictor	Outcome	Spearman *r*	Spearman *p*	Spearman *q*
Combined	FAI	E-selectin	0.442	0.002	0.042
PMOS	FAI	E-selectin	0.72	<0.0001	0.009
PMOS	FAI	1,25(OH)_2_D_3_	0.708	0.001	0.018

## Data Availability

The original contributions presented in this study are included in the article/Supplementary Materials. Further inquiries can be directed to the corresponding author.

## Supplementary Materials

The following supporting information can be downloaded at: https://www.mdpi.com/article/10.3390/ijms27177661/s1.

## Abbreviations

BH-FDR, Benjamini–Hochberg false discovery rate; CI, confidence interval; ICAM, intercellular adhesion molecule; IL, interleukin; PAI-1, plasminogen activator inhibitor-1; PMOS, polyendocrine metabolic ovarian syndrome; RFU, relative fluorescence units; SDF-1, stromal cell-derived factor-1; TNF, tumour necrosis factor; tPA, tissue-type plasminogen activator; VCAM-1, vascular cell adhesion molecule-1; VEGF-A, vascular endothelial growth factor-A; vWF, von Willebrand factor.
