# Supplementary material for: A Cross-Sectional Exploratory Study of Endothelial Biomarkers in Non-Obese Women with and Without Polyendocrine Metabolic Ovarian Syndrome
Source: Int J Mol Sci. 2026 Aug 26;27(17):7661. doi: 10.3390/ijms27177661 (PMC13566641; doi:10.3390/ijms27177661)
Supplement: Supplementary file 1 [file ijms-27-07661-s001.zip › Table S6 conttrol FAI HOMA AMH_aeb.pdf]

Supplementary Table S6. Complete Pearson correlation analyses of HOMA-IR, FAI and AMH with vitamin D metabolites and inflammatory/endothelial biomarkers in the control cohort

Benjamini–Hochberg false-discovery-rate correction was applied within each predefined family of correlations. Pearson r denotes the Pearson correlation coefficient; p denotes the unadjusted two-sided p value; q denotes the BH-FDR-adjusted p value. q<0.05 was considered statistically significant.

Predictor	Outcome	Category	Pearson r	Pearson p	Pearson q (BH-FDR)
HOMA-IR	$1,25(OH)_2D_3$	Vitamin D	-0.019	0.935	0.974
HOMA-IR	$25(OH)D_3$	Vitamin D	-0.195	0.321	0.846
HOMA-IR	$25(OH)D_2$	Vitamin D	-0.182	0.394	0.846
HOMA-IR	$24,25(OH)_2D_3$	Vitamin D	-0.106	0.590	0.931
HOMA-IR	E-selectin	Inflammatory/endothelial	-0.043	0.828	0.931
HOMA-IR	ICAM-1	Inflammatory/endothelial	-0.148	0.451	0.846
HOMA-IR	VCAM-1	Inflammatory/endothelial	-0.163	0.406	0.846
HOMA-IR	Cadherin-5	Inflammatory/endothelial	-0.467	0.012	0.439
HOMA-IR	P-selectin	Inflammatory/endothelial	0.738	<0.001	**0.001**
HOMA-IR	von Willebrand factor	Inflammatory/endothelial	-0.087	0.661	0.931
HOMA-IR	ICAM-3	Inflammatory/endothelial	0.301	0.120	0.751
HOMA-IR	ICAM-5	Inflammatory/endothelial	-0.258	0.184	0.751
HOMA-IR	ICAM-2	Inflammatory/endothelial	0.411	0.030	0.439
HOMA-IR	VEGF-A	Inflammatory/endothelial	-0.268	0.167	0.751
HOMA-IR	Angiopoietin-1	Inflammatory/endothelial	0.134	0.495	0.884
HOMA-IR	Angiopoietin-2	Inflammatory/endothelial	-0.250	0.200	0.751
HOMA-IR	Soluble Tie-2	Inflammatory/endothelial	-0.417	0.027	0.439
HOMA-IR	TNF	Inflammatory/endothelial	0.049	0.805	0.931
HOMA-IR	IL-1α	Inflammatory/endothelial	0.226	0.247	0.805
HOMA-IR	IL-6	Inflammatory/endothelial	-0.007	0.971	0.981
HOMA-IR	Tissue Factor	Inflammatory/endothelial	-0.283	0.144	0.751

HOMA-IR	tPA	Inflammatory/endothelial	0.250	0.199	0.751
HOMA-IR	PAI-1	Inflammatory/endothelial	0.407	0.032	0.439
HOMA-IR	D-dimer	Inflammatory/endothelial	-0.163	0.408	0.846
HOMA-IR	SDF-1	Inflammatory/endothelial	-0.199	0.310	0.846
FAI	$1,25(OH)_2D_3$	Vitamin D	-0.310	0.183	0.751
FAI	$25(OH)D_3$	Vitamin D	-0.010	0.958	0.981
FAI	$25(OH)D_2$	Vitamin D	-0.189	0.377	0.846
FAI	$24,25(OH)_2D_3$	Vitamin D	0.128	0.516	0.901
FAI	E-selectin	Inflammatory/endothelial	0.228	0.244	0.805
FAI	ICAM-1	Inflammatory/endothelial	0.221	0.258	0.805
FAI	VCAM-1	Inflammatory/endothelial	0.372	0.051	0.482
FAI	Cadherin-5	Inflammatory/endothelial	0.395	0.037	0.439
FAI	P-selectin	Inflammatory/endothelial	0.045	0.819	0.931
FAI	von Willebrand factor	Inflammatory/endothelial	0.275	0.157	0.751
FAI	ICAM-3	Inflammatory/endothelial	0.140	0.477	0.873
FAI	ICAM-5	Inflammatory/endothelial	-0.156	0.427	0.846
FAI	ICAM-2	Inflammatory/endothelial	0.089	0.652	0.931
FAI	VEGF-A	Inflammatory/endothelial	-0.150	0.445	0.846
FAI	Angiopoietin-1	Inflammatory/endothelial	0.162	0.410	0.846
FAI	Angiopoietin-2	Inflammatory/endothelial	0.215	0.271	0.812
FAI	Soluble Tie-2	Inflammatory/endothelial	0.032	0.871	0.933
FAI	TNF	Inflammatory/endothelial	-0.041	0.836	0.931
FAI	IL-1α	Inflammatory/endothelial	-0.177	0.368	0.846
FAI	IL-6	Inflammatory/endothelial	0.151	0.442	0.846
FAI	Tissue Factor	Inflammatory/endothelial	-0.107	0.588	0.931
FAI	tPA	Inflammatory/endothelial	0.239	0.221	0.788
FAI	PAI-1	Inflammatory/endothelial	-0.079	0.690	0.931
FAI	D-dimer	Inflammatory/endothelial	0.334	0.082	0.615
FAI	SDF-1	Inflammatory/endothelial	-0.343	0.074	0.614
AMH	$1,25(OH)_2D_3$	Vitamin D	-0.053	0.823	0.931

AMH	25(OH)D_3	Vitamin D	-0.084	0.671	0.931
AMH	25(OH)D_2	Vitamin D	-0.219	0.303	0.846
AMH	24,25$(OH)_2D_3$	Vitamin D	0.057	0.775	0.931
AMH	E-selectin	Inflammatory/endothelial	-0.005	0.981	0.981
AMH	ICAM-1	Inflammatory/endothelial	-0.110	0.578	0.931
AMH	VCAM-1	Inflammatory/endothelial	0.071	0.719	0.931
AMH	Cadherin-5	Inflammatory/endothelial	0.268	0.168	0.751
AMH	P-selectin	Inflammatory/endothelial	-0.091	0.647	0.931
AMH	von Willebrand factor	Inflammatory/endothelial	0.086	0.662	0.931
AMH	ICAM-3	Inflammatory/endothelial	0.029	0.885	0.935
AMH	ICAM-5	Inflammatory/endothelial	0.057	0.775	0.931
AMH	ICAM-2	Inflammatory/endothelial	-0.039	0.843	0.931
AMH	VEGF-A	Inflammatory/endothelial	-0.109	0.581	0.931
AMH	Angiopoietin-1	Inflammatory/endothelial	0.174	0.376	0.846
AMH	Angiopoietin-2	Inflammatory/endothelial	0.389	0.041	0.439
AMH	Soluble Tie-2	Inflammatory/endothelial	0.032	0.871	0.933
AMH	TNF	Inflammatory/endothelial	0.265	0.173	0.751
AMH	IL-1α	Inflammatory/endothelial	0.072	0.714	0.931
AMH	IL-6	Inflammatory/endothelial	-0.039	0.844	0.931
AMH	Tissue Factor	Inflammatory/endothelial	-0.083	0.673	0.931
AMH	tPA	Inflammatory/endothelial	-0.048	0.809	0.931
AMH	PAI-1	Inflammatory/endothelial	-0.047	0.811	0.931
AMH	D-dimer	Inflammatory/endothelial	0.158	0.421	0.846
AMH	SDF-1	Inflammatory/endothelial	-0.056	0.778	0.931
