# Supplementary material for: A Cross-Sectional Exploratory Study of Endothelial Biomarkers in Non-Obese Women with and Without Polyendocrine Metabolic Ovarian Syndrome
Source: Int J Mol Sci. 2026 Aug 26;27(17):7661. doi: 10.3390/ijms27177661 (PMC13566641; doi:10.3390/ijms27177661)
Supplement: Supplementary file 1 [file ijms-27-07661-s001.zip › Table S1 Combined correlations between inflammatory_aeb.pdf]

Supplementary Table S1. Correlations between inflammatory/endothelial markers and vitamin D metabolites — Combined

Benjamini–Hochberg false-discovery-rate correction was applied within each predefined family of correlations. Pearson r denotes the Pearson correlation coefficient; p denotes the unadjusted sided p value; q denotes the BH-FDR-adjusted p value. q<0.05 was considered statistically significant.

**Vitamin D metabolite**	**Marker**	**Pearson r**	**Pearson p**	**Pearson BH-FDR q**
1,25$(OH)_2D_3$	E-selectin	0.008	0.961	0.961
1,25$(OH)_2D_3$	Intercellular adhesion molecule 1	-0.017	0.919	0.961
1,25$(OH)_2D_3$	Vascular cell adhesion protein 1	-0.167	0.324	0.961
1,25$(OH)_2D_3$	Cadherin-5	-0.084	0.623	0.961
1,25$(OH)_2D_3$	P-selectin	0.019	0.909	0.961
1,25$(OH)_2D_3$	von Willebrand factor	-0.069	0.683	0.961
1,25$(OH)_2D_3$	Intercellular adhesion molecule 3	-0.016	0.927	0.961
1,25$(OH)_2D_3$	Intercellular adhesion molecule 5	-0.089	0.599	0.961
1,25$(OH)_2D_3$	Intercellular adhesion molecule 2	-0.088	0.605	0.961
1,25$(OH)_2D_3$	Vascular endothelial growth factor A	0.042	0.803	0.961
1,25$(OH)_2D_3$	Angiopoietin-1	0.027	0.873	0.961
1,25$(OH)_2D_3$	Angiopoietin-2	-0.091	0.591	0.961
1,25$(OH)_2D_3$	Angiopoietin-1 receptor, soluble	-0.171	0.313	0.961
1,25$(OH)_2D_3$	Tumor necrosis factor	-0.056	0.741	0.961
1,25$(OH)_2D_3$	Interleukin-1 alpha	0.246	0.142	0.961
1,25$(OH)_2D_3$	Interleukin-6	-0.026	0.877	0.961
1,25$(OH)_2D_3$	Tissue Factor	-0.191	0.257	0.961
1,25$(OH)_2D_3$	Tissue-type plasminogen activator	0.128	0.449	0.961
1,25$(OH)_2D_3$	Plasminogen activator inhibitor 1	0.175	0.301	0.961
1,25$(OH)_2D_3$	D-dimer	-0.015	0.932	0.961
1,25$(OH)_2D_3$	Stromal cell-derived factor 1	-0.108	0.524	0.961
25$(OH)D_3$	E-selectin	0.176	0.231	0.969
25$(OH)D_3$	Intercellular adhesion molecule 1	-0.005	0.972	0.997
25$(OH)D_3$	Vascular cell adhesion protein 1	0.038	0.797	0.997
25$(OH)D_3$	Cadherin-5	0.024	0.874	0.997

25(OH)D_3	P-selectin	-0.238	0.104	0.913
25(OH)D_3	von Willebrand factor	-0.001	0.997	0.997
25(OH)D_3	Intercellular adhesion molecule 3	0.036	0.810	0.997
25(OH)D_3	Intercellular adhesion molecule 5	0.075	0.613	0.997
25(OH)D_3	Intercellular adhesion molecule 2	0.004	0.980	0.997
25(OH)D_3	Vascular endothelial growth factor A	0.035	0.812	0.997
25(OH)D_3	Angiopoietin-1	-0.107	0.468	0.997
25(OH)D_3	Angiopoietin-2	-0.005	0.974	0.997
25(OH)D_3	Angiopoietin-1 receptor, soluble	-0.068	0.645	0.997
25(OH)D_3	Tumor necrosis factor	0.032	0.829	0.997
25(OH)D_3	Interleukin-1 alpha	0.035	0.815	0.997
25(OH)D_3	Interleukin-6	-0.232	0.112	0.913
25(OH)D_3	Tissue Factor	-0.121	0.413	0.997
25(OH)D_3	Tissue-type plasminogen activator	0.030	0.841	0.997
25(OH)D_3	Plasminogen activator inhibitor 1	-0.180	0.220	0.969
25(OH)D_3	D-dimer	-0.018	0.903	0.997
25(OH)D_3	Stromal cell-derived factor 1	-0.221	0.130	0.913
25(OH)D_2	E-selectin	0.014	0.937	0.984
25(OH)D_2	Intercellular adhesion molecule 1	0.049	0.775	0.984
25(OH)D_2	Vascular cell adhesion protein 1	-0.165	0.330	0.984
25(OH)D_2	Cadherin-5	0.010	0.954	0.984
25(OH)D_2	P-selectin	-0.083	0.625	0.984
25(OH)D_2	von Willebrand factor	0.074	0.664	0.984
25(OH)D_2	Intercellular adhesion molecule 3	-0.131	0.439	0.984
25(OH)D_2	Intercellular adhesion molecule 5	0.181	0.284	0.984
25(OH)D_2	Intercellular adhesion molecule 2	-0.137	0.419	0.984
25(OH)D_2	Vascular endothelial growth factor A	0.076	0.656	0.984
25(OH)D_2	Angiopoietin-1	-0.154	0.363	0.984
25(OH)D_2	Angiopoietin-2	-0.062	0.717	0.984
25(OH)D_2	Angiopoietin-1 receptor, soluble	0.106	0.532	0.984
25(OH)D_2	Tumor necrosis factor	-0.030	0.861	0.984
25(OH)D_2	Interleukin-1 alpha	-0.003	0.984	0.984

$25(OH)D_2$	Interleukin-6	-0.027	0.874	0.984
$25(OH)D_2$	Tissue Factor	0.112	0.508	0.984
$25(OH)D_2$	Tissue-type plasminogen activator	-0.064	0.709	0.984
$25(OH)D_2$	Plasminogen activator inhibitor 1	-0.137	0.418	0.984
$25(OH)D_2$	D-dimer	0.036	0.833	0.984
$25(OH)D_2$	Stromal cell-derived factor 1	-0.079	0.644	0.984
$24,25(OH)_2D_3$	E-selectin	0.179	0.222	0.934
$24,25(OH)_2D_3$	Intercellular adhesion molecule 1	0.003	0.984	0.985
$24,25(OH)_2D_3$	Vascular cell adhesion protein 1	0.132	0.370	0.985
$24,25(OH)_2D_3$	Cadherin-5	0.092	0.534	0.985
$24,25(OH)_2D_3$	P-selectin	-0.273	0.060	0.779
$24,25(OH)_2D_3$	von Willebrand factor	-0.056	0.705	0.985
$24,25(OH)_2D_3$	Intercellular adhesion molecule 3	0.016	0.912	0.985
$24,25(OH)_2D_3$	Intercellular adhesion molecule 5	0.069	0.640	0.985
$24,25(OH)_2D_3$	Intercellular adhesion molecule 2	-0.019	0.900	0.985
$24,25(OH)_2D_3$	Vascular endothelial growth factor A	0.017	0.908	0.985
$24,25(OH)_2D_3$	Angiopoietin-1	-0.212	0.148	0.779
$24,25(OH)_2D_3$	Angiopoietin-2	0.003	0.985	0.985
$24,25(OH)_2D_3$	Angiopoietin-1 receptor, soluble	-0.035	0.813	0.985
$24,25(OH)_2D_3$	Tumor necrosis factor	0.119	0.421	0.985
$24,25(OH)_2D_3$	Interleukin-1 alpha	0.093	0.531	0.985
$24,25(OH)_2D_3$	Interleukin-6	-0.154	0.296	0.985
$24,25(OH)_2D_3$	Tissue Factor	-0.070	0.635	0.985
$24,25(OH)_2D_3$	Tissue-type plasminogen activator	0.008	0.957	0.985
$24,25(OH)_2D_3$	Plasminogen activator inhibitor 1	-0.247	0.091	0.779
$24,25(OH)_2D_3$	D-dimer	-0.058	0.696	0.985
$24,25(OH)_2D_3$	Stromal cell-derived factor 1	-0.221	0.132	0.779
