# Supplementary material for: A Cross-Sectional Exploratory Study of Endothelial Biomarkers in Non-Obese Women with and Without Polyendocrine Metabolic Ovarian Syndrome
Source: Int J Mol Sci. 2026 Aug 26;27(17):7661. doi: 10.3390/ijms27177661 (PMC13566641; doi:10.3390/ijms27177661)
Supplement: Supplementary file 1 [file ijms-27-07661-s001.zip › Table S2 PMOS Correlations between inflammatory_aeb.pdf]

Supplementary Table S2. Correlations between inflammatory/endothelial markers and vitamin D metabolites — PMOS

Benjamini–Hochberg false-discovery-rate correction was applied within each predefined family of correlations. Pearson r denotes the Pearson correlation coefficient; p denotes the unadjusted two-sided p value; q denotes the BH-FDR-adjusted p value. q<0.05 was considered statistically significant.

Vitamin D metabolite	Marker	Pearson r	Pearson p	Pearson BH-FDR q
$1,25(OH)_2D_3$	E-selectin	0.338	0.185	0.939
$1,25(OH)_2D_3$	Intercellular adhesion molecule 1	0.444	0.074	0.939
$1,25(OH)_2D_3$	Vascular cell adhesion protein 1	0.150	0.567	0.939
$1,25(OH)_2D_3$	Cadherin-5	0.258	0.318	0.939
$1,25(OH)_2D_3$	P-selectin	0.040	0.880	0.948
$1,25(OH)_2D_3$	von Willebrand factor	-0.080	0.760	0.939
$1,25(OH)_2D_3$	Intercellular adhesion molecule 3	0.223	0.389	0.939
$1,25(OH)_2D_3$	Intercellular adhesion molecule 5	-0.104	0.690	0.939
$1,25(OH)_2D_3$	Intercellular adhesion molecule 2	0.293	0.254	0.939
$1,25(OH)_2D_3$	Vascular endothelial growth factor A	-0.039	0.882	0.948
$1,25(OH)_2D_3$	Angiopoietin-1	-0.145	0.579	0.939
$1,25(OH)_2D_3$	Angiopoietin-2	0.223	0.390	0.939
$1,25(OH)_2D_3$	Angiopoietin-1 receptor, soluble	-0.108	0.680	0.939
$1,25(OH)_2D_3$	Tumor necrosis factor	0.289	0.261	0.939
$1,25(OH)_2D_3$	Interleukin-1 alpha	0.214	0.409	0.939
$1,25(OH)_2D_3$	Interleukin-6	0.029	0.912	0.948
$1,25(OH)_2D_3$	Tissue Factor	0.196	0.451	0.939
$1,25(OH)_2D_3$	Tissue-type plasminogen activator	0.420	0.094	0.939

1,25$(OH)_2D_3$	Plasminogen activator inhibitor 1	0.087	0.740	0.939
1,25$(OH)_2D_3$	D-dimer	0.017	0.948	0.948
1,25$(OH)_2D_3$	Stromal cell-derived factor 1	-0.082	0.754	0.939
25$(OH)D_3$	E-selectin	0.417	0.067	0.295
25$(OH)D_3$	Intercellular adhesion molecule 1	-0.060	0.803	0.887
25$(OH)D_3$	Vascular cell adhesion protein 1	0.166	0.485	0.599
25$(OH)D_3$	Cadherin-5	0.358	0.121	0.364
25$(OH)D_3$	P-selectin	-0.276	0.239	0.456
25$(OH)D_3$	von Willebrand factor	-0.232	0.326	0.570
25$(OH)D_3$	Intercellular adhesion molecule 3	0.167	0.482	0.599
25$(OH)D_3$	Intercellular adhesion molecule 5	0.176	0.458	0.599
25$(OH)D_3$	Intercellular adhesion molecule 2	0.317	0.174	0.406
25$(OH)D_3$	Vascular endothelial growth factor A	0.023	0.925	0.971
25$(OH)D_3$	Angiopoietin-1	-0.497	0.026	0.238
25$(OH)D_3$	Angiopoietin-2	0.375	0.103	0.361
25$(OH)D_3$	Angiopoietin-1 receptor, soluble	0.174	0.462	0.599
25$(OH)D_3$	Tumor necrosis factor	0.561	0.010	0.213
25$(OH)D_3$	Interleukin-1 alpha	0.325	0.162	0.406
25$(OH)D_3$	Interleukin-6	-0.209	0.377	0.599
25$(OH)D_3$	Tissue Factor	0.105	0.660	0.770
25$(OH)D_3$	Tissue-type plasminogen activator	0.004	0.986	0.986
25$(OH)D_3$	Plasminogen activator inhibitor 1	-0.413	0.070	0.295
25$(OH)D_3$	D-dimer	-0.285	0.223	0.456
25$(OH)D_3$	Stromal cell-derived factor 1	-0.476	0.034	0.238
25$(OH)D_2$	E-selectin	0.287	0.343	0.860

25(OH)D_2	Intercellular adhesion molecule 1	-0.100	0.745	0.885
25(OH)D_2	Vascular cell adhesion protein 1	0.095	0.759	0.885
25(OH)D_2	Cadherin-5	0.230	0.450	0.860
25(OH)D_2	P-selectin	0.391	0.186	0.860
25(OH)D_2	von Willebrand factor	0.117	0.704	0.885
25(OH)D_2	Intercellular adhesion molecule 3	-0.423	0.150	0.860
25(OH)D_2	Intercellular adhesion molecule 5	-0.125	0.685	0.885
25(OH)D_2	Intercellular adhesion molecule 2	-0.263	0.386	0.860
25(OH)D_2	Vascular endothelial growth factor A	-0.078	0.801	0.885
25(OH)D_2	Angiopoietin-1	0.120	0.697	0.885
25(OH)D_2	Angiopoietin-2	-0.236	0.439	0.860
25(OH)D_2	Angiopoietin-1 receptor, soluble	0.230	0.449	0.860
25(OH)D_2	Tumor necrosis factor	0.127	0.680	0.885
25(OH)D_2	Interleukin-1 alpha	-0.026	0.933	0.933
25(OH)D_2	Interleukin-6	-0.210	0.492	0.861
25(OH)D_2	Tissue Factor	0.489	0.090	0.860
25(OH)D_2	Tissue-type plasminogen activator	0.373	0.209	0.860
25(OH)D_2	Plasminogen activator inhibitor 1	0.348	0.245	0.860
25(OH)D_2	D-dimer	0.230	0.450	0.860
25(OH)D_2	Stromal cell-derived factor 1	-0.040	0.897	0.933
24,25$(OH)_2D_3$	E-selectin	0.245	0.297	0.517
24,25$(OH)_2D_3$	Intercellular adhesion molecule 1	-0.157	0.510	0.713
24,25$(OH)_2D_3$	Vascular cell adhesion protein 1	0.168	0.478	0.713
24,25$(OH)_2D_3$	Cadherin-5	0.250	0.287	0.517
24,25$(OH)_2D_3$	P-selectin	-0.328	0.158	0.415

24,25$(OH)_2D_3$	von Willebrand factor	-0.270	0.250	0.517
24,25$(OH)_2D_3$	Intercellular adhesion molecule 3	0.234	0.320	0.517
24,25$(OH)_2D_3$	Intercellular adhesion molecule 5	0.116	0.627	0.813
24,25$(OH)_2D_3$	Intercellular adhesion molecule 2	0.360	0.119	0.358
24,25$(OH)_2D_3$	Vascular endothelial growth factor A	-0.106	0.658	0.813
24,25$(OH)_2D_3$	Angiopoietin-1	-0.505	0.023	0.166
24,25$(OH)_2D_3$	Angiopoietin-2	0.365	0.113	0.358
24,25$(OH)_2D_3$	Angiopoietin-1 receptor, soluble	0.024	0.920	0.920
24,25$(OH)_2D_3$	Tumor necrosis factor	0.648	0.002	**0.042**
24,25$(OH)_2D_3$	Interleukin-1 alpha	0.368	0.110	0.358
24,25$(OH)_2D_3$	Interleukin-6	-0.069	0.773	0.836
24,25$(OH)_2D_3$	Tissue Factor	0.063	0.792	0.836
24,25$(OH)_2D_3$	Tissue-type plasminogen activator	-0.062	0.796	0.836
24,25$(OH)_2D_3$	Plasminogen activator inhibitor 1	-0.405	0.077	0.358
24,25$(OH)_2D_3$	D-dimer	-0.302	0.195	0.455
24,25$(OH)_2D_3$	Stromal cell-derived factor 1	-0.503	0.024	0.166
