# Supplementary material for: A Cross-Sectional Exploratory Study of Endothelial Biomarkers in Non-Obese Women with and Without Polyendocrine Metabolic Ovarian Syndrome
Source: Int J Mol Sci. 2026 Aug 26;27(17):7661. doi: 10.3390/ijms27177661 (PMC13566641; doi:10.3390/ijms27177661)
Supplement: Supplementary file 1 [file ijms-27-07661-s001.zip › Table S3 Controls correlations between inflammatory_aeb.pdf]

Supplementary Table S3. Correlations between inflammatory/endothelial markers and vitamin D metabolites — Controls

Benjamini–Hochberg false-discovery-rate correction was applied within each predefined family of correlations. Pearson r denotes the Pearson correlation coefficient; p denotes the unadjusted two-sided p value; q denotes the BH-FDR-adjusted p value. q<0.05 was considered statistically significant.

Vitamin D metabolite	Marker	Pearson r	Pearson p	Pearson BH-FDR q
$1,25(OH)_2D_3$	E-selectin	-0.251	0.287	0.901
$1,25(OH)_2D_3$	Intercellular adhesion molecule 1	-0.373	0.106	0.901
$1,25(OH)_2D_3$	Vascular cell adhesion protein 1	-0.409	0.073	0.901
$1,25(OH)_2D_3$	Cadherin-5	-0.258	0.272	0.901
$1,25(OH)_2D_3$	P-selectin	0.002	0.992	0.992
$1,25(OH)_2D_3$	von Willebrand factor	-0.060	0.803	0.959
$1,25(OH)_2D_3$	Intercellular adhesion molecule 3	-0.064	0.789	0.959
$1,25(OH)_2D_3$	Intercellular adhesion molecule 5	-0.072	0.762	0.959
$1,25(OH)_2D_3$	Intercellular adhesion molecule 2	-0.143	0.548	0.959
$1,25(OH)_2D_3$	Vascular endothelial growth factor A	0.054	0.822	0.959
$1,25(OH)_2D_3$	Angiopoietin-1	0.110	0.644	0.959
$1,25(OH)_2D_3$	Angiopoietin-2	-0.223	0.345	0.901
$1,25(OH)_2D_3$	Angiopoietin-1 receptor, soluble	-0.205	0.386	0.901
$1,25(OH)_2D_3$	Tumor necrosis factor	-0.168	0.478	0.959
$1,25(OH)_2D_3$	Interleukin-1 alpha	0.272	0.247	0.901
$1,25(OH)_2D_3$	Interleukin-6	-0.109	0.647	0.959
$1,25(OH)_2D_3$	Tissue Factor	-0.317	0.173	0.901
$1,25(OH)_2D_3$	Tissue-type plasminogen activator	-0.034	0.887	0.980
$1,25(OH)_2D_3$	Plasminogen activator inhibitor 1	0.239	0.310	0.901
$1,25(OH)_2D_3$	D-dimer	-0.016	0.945	0.992
$1,25(OH)_2D_3$	Stromal cell-derived factor 1	-0.114	0.632	0.959
$25(OH)D_3$	E-selectin	-0.009	0.962	0.968
$25(OH)D_3$	Intercellular adhesion molecule 1	0.041	0.835	0.968
$25(OH)D_3$	Vascular cell adhesion protein 1	-0.052	0.793	0.968
$25(OH)D_3$	Cadherin-5	-0.189	0.336	0.968

$25(OH)D_3$	P-selectin	-0.212	0.279	0.968
$25(OH)D_3$	von Willebrand factor	0.111	0.574	0.968
$25(OH)D_3$	Intercellular adhesion molecule 3	0.008	0.968	0.968
$25(OH)D_3$	Intercellular adhesion molecule 5	0.034	0.864	0.968
$25(OH)D_3$	Intercellular adhesion molecule 2	-0.049	0.804	0.968
$25(OH)D_3$	Vascular endothelial growth factor A	0.037	0.853	0.968
$25(OH)D_3$	Angiopoietin-1	0.072	0.715	0.968
$25(OH)D_3$	Angiopoietin-2	-0.174	0.376	0.968
$25(OH)D_3$	Angiopoietin-1 receptor, soluble	-0.228	0.244	0.968
$25(OH)D_3$	Tumor necrosis factor	-0.158	0.421	0.968
$25(OH)D_3$	Interleukin-1 alpha	-0.098	0.621	0.968
$25(OH)D_3$	Interleukin-6	-0.306	0.114	0.968
$25(OH)D_3$	Tissue Factor	-0.201	0.304	0.968
$25(OH)D_3$	Tissue-type plasminogen activator	0.044	0.824	0.968
$25(OH)D_3$	Plasminogen activator inhibitor 1	-0.019	0.925	0.968
$25(OH)D_3$	D-dimer	0.060	0.762	0.968
$25(OH)D_3$	Stromal cell-derived factor 1	-0.070	0.722	0.968
$25(OH)D_2$	E-selectin	0.025	0.907	0.968
$25(OH)D_2$	Intercellular adhesion molecule 1	0.103	0.632	0.968
$25(OH)D_2$	Vascular cell adhesion protein 1	-0.170	0.427	0.968
$25(OH)D_2$	Cadherin-5	-0.027	0.902	0.968
$25(OH)D_2$	P-selectin	-0.204	0.339	0.968
$25(OH)D_2$	von Willebrand factor	0.048	0.823	0.968
$25(OH)D_2$	Intercellular adhesion molecule 3	-0.129	0.549	0.968
$25(OH)D_2$	Intercellular adhesion molecule 5	0.226	0.289	0.968
$25(OH)D_2$	Intercellular adhesion molecule 2	-0.185	0.386	0.968
$25(OH)D_2$	Vascular endothelial growth factor A	0.166	0.438	0.968
$25(OH)D_2$	Angiopoietin-1	-0.314	0.135	0.968
$25(OH)D_2$	Angiopoietin-2	-0.018	0.933	0.968
$25(OH)D_2$	Angiopoietin-1 receptor, soluble	0.145	0.499	0.968
$25(OH)D_2$	Tumor necrosis factor	-0.049	0.821	0.968
$25(OH)D_2$	Interleukin-1 alpha	-0.019	0.930	0.968

$25(OH)D_2$	Interleukin-6	0.009	0.968	0.968
$25(OH)D_2$	Tissue Factor	0.069	0.748	0.968
$25(OH)D_2$	Tissue-type plasminogen activator	-0.136	0.526	0.968
$25(OH)D_2$	Plasminogen activator inhibitor 1	-0.307	0.145	0.968
$25(OH)D_2$	D-dimer	-0.009	0.965	0.968
$25(OH)D_2$	Stromal cell-derived factor 1	-0.152	0.478	0.968
$24,25(OH)_2D_3$	E-selectin	0.122	0.537	0.961
$24,25(OH)_2D_3$	Intercellular adhesion molecule 1	0.153	0.436	0.961
$24,25(OH)_2D_3$	Vascular cell adhesion protein 1	0.107	0.587	0.961
$24,25(OH)_2D_3$	Cadherin-5	-0.010	0.961	0.961
$24,25(OH)_2D_3$	P-selectin	-0.224	0.253	0.961
$24,25(OH)_2D_3$	von Willebrand factor	0.059	0.765	0.961
$24,25(OH)_2D_3$	Intercellular adhesion molecule 3	-0.037	0.851	0.961
$24,25(OH)_2D_3$	Intercellular adhesion molecule 5	0.065	0.741	0.961
$24,25(OH)_2D_3$	Intercellular adhesion molecule 2	-0.088	0.657	0.961
$24,25(OH)_2D_3$	Vascular endothelial growth factor A	0.053	0.788	0.961
$24,25(OH)_2D_3$	Angiopoietin-1	-0.060	0.760	0.961
$24,25(OH)_2D_3$	Angiopoietin-2	-0.175	0.373	0.961
$24,25(OH)_2D_3$	Angiopoietin-1 receptor, soluble	-0.087	0.660	0.961
$24,25(OH)_2D_3$	Tumor necrosis factor	-0.085	0.669	0.961
$24,25(OH)_2D_3$	Interleukin-1 alpha	-0.040	0.839	0.961
$24,25(OH)_2D_3$	Interleukin-6	-0.310	0.108	0.961
$24,25(OH)_2D_3$	Tissue Factor	-0.121	0.540	0.961
$24,25(OH)_2D_3$	Tissue-type plasminogen activator	0.052	0.794	0.961
$24,25(OH)_2D_3$	Plasminogen activator inhibitor 1	-0.121	0.539	0.961
$24,25(OH)_2D_3$	D-dimer	0.021	0.916	0.961
$24,25(OH)_2D_3$	Stromal cell-derived factor 1	-0.031	0.875	0.961
