# Supplementary material for: A Cross-Sectional Exploratory Study of Endothelial Biomarkers in Non-Obese Women with and Without Polyendocrine Metabolic Ovarian Syndrome
Source: Int J Mol Sci. 2026 Aug 26;27(17):7661. doi: 10.3390/ijms27177661 (PMC13566641; doi:10.3390/ijms27177661)
Supplement: Supplementary file 1 [file ijms-27-07661-s001.zip › Table S4 combined FAI HOMA AMH_aeb.pdf]

Supplementary Table S4. Complete Pearson correlation analyses of HOMA-IR, FAI and AMH with vitamin D metabolites and inflammatory/endothelial biomarkers in the combined cohort

Benjamini–Hochberg false-discovery-rate correction was applied within each predefined family of correlations. Pearson r denotes the Pearson correlation coefficient; p denotes the unadjusted two-sided p value; q denotes the BH-FDR-adjusted p value. q<0.05 was considered statistically significant.

Predictor	Outcome	Category	Pearson r	Pearson p	Pearson q (BH-FDR)
HOMA-IR	$1,25(OH)_2D_3$	Vitamin D	0.094	0.581	0.955
HOMA-IR	$25(OH)D_3$	Vitamin D	-0.046	0.758	0.972
HOMA-IR	$25(OH)D_2$	Vitamin D	-0.116	0.493	0.955
HOMA-IR	$24,25(OH)_2D_3$	Vitamin D	0.019	0.897	0.972
HOMA-IR	E-selectin	Inflammatory/endothelial	0.044	0.768	0.972
HOMA-IR	ICAM-1	Inflammatory/endothelial	0.005	0.972	0.972
HOMA-IR	VCAM-1	Inflammatory/endothelial	-0.194	0.187	0.778
HOMA-IR	Cadherin-5	Inflammatory/endothelial	-0.372	0.009	0.158
HOMA-IR	P-selectin	Inflammatory/endothelial	0.502	<0.001	**0.021**
HOMA-IR	von Willebrand factor	Inflammatory/endothelial	-0.042	0.779	0.972
HOMA-IR	ICAM-3	Inflammatory/endothelial	0.264	0.070	0.398
HOMA-IR	ICAM-5	Inflammatory/endothelial	-0.273	0.060	0.398
HOMA-IR	ICAM-2	Inflammatory/endothelial	0.436	0.002	**0.049**
HOMA-IR	VEGF-A	Inflammatory/endothelial	-0.262	0.072	0.398
HOMA-IR	Angiopoietin-1	Inflammatory/endothelial	0.137	0.354	0.937
HOMA-IR	Angiopoietin-2	Inflammatory/endothelial	-0.251	0.086	0.429
HOMA-IR	Soluble Tie-2	Inflammatory/endothelial	-0.366	0.011	0.158
HOMA-IR	TNF	Inflammatory/endothelial	0.082	0.577	0.955
HOMA-IR	IL-1α	Inflammatory/endothelial	0.278	0.056	0.398
HOMA-IR	IL-6	Inflammatory/endothelial	-0.029	0.846	0.972
HOMA-IR	Tissue Factor	Inflammatory/endothelial	-0.305	0.035	0.326

HOMA-IR	tPA	Inflammatory/endothelial	0.260	0.074	0.398
HOMA-IR	PAI-1	Inflammatory/endothelial	0.326	0.024	0.295
HOMA-IR	D-dimer	Inflammatory/endothelial	-0.091	0.540	0.955
HOMA-IR	SDF-1	Inflammatory/endothelial	-0.208	0.156	0.689
FAI	1α,25(OH)$_2$D$_3$	Vitamin D	0.251	0.134	0.628
FAI	25(OH)D$_3$	Vitamin D	0.134	0.362	0.937
FAI	25(OH)D$_2$	Vitamin D	-0.186	0.270	0.899
FAI	24R,25(OH)$_2$D$_3$	Vitamin D	0.166	0.260	0.899
FAI	E-selectin	Inflammatory/endothelial	0.439	0.002	**0.049**
FAI	ICAM-1	Inflammatory/endothelial	0.187	0.202	0.798
FAI	VCAM-1	Inflammatory/endothelial	0.100	0.499	0.955
FAI	Cadherin-5	Inflammatory/endothelial	0.058	0.697	0.969
FAI	P-selectin	Inflammatory/endothelial	0.071	0.631	0.955
FAI	von Willebrand factor	Inflammatory/endothelial	-0.074	0.615	0.955
FAI	ICAM-3	Inflammatory/endothelial	0.012	0.935	0.972
FAI	ICAM-5	Inflammatory/endothelial	-0.064	0.668	0.963
FAI	ICAM-2	Inflammatory/endothelial	0.007	0.963	0.972
FAI	VEGF-A	Inflammatory/endothelial	0.089	0.546	0.955
FAI	Angiopoietin-1	Inflammatory/endothelial	-0.151	0.307	0.920
FAI	Angiopoietin-2	Inflammatory/endothelial	0.101	0.493	0.955
FAI	Soluble Tie-2	Inflammatory/endothelial	-0.024	0.870	0.972
FAI	TNF	Inflammatory/endothelial	0.059	0.691	0.969
FAI	IL-1α	Inflammatory/endothelial	-0.067	0.649	0.955
FAI	IL-6	Inflammatory/endothelial	0.026	0.860	0.972
FAI	Tissue Factor	Inflammatory/endothelial	-0.006	0.967	0.972
FAI	tPA	Inflammatory/endothelial	0.144	0.329	0.936
FAI	PAI-1	Inflammatory/endothelial	-0.107	0.467	0.955
FAI	D-dimer	Inflammatory/endothelial	-0.051	0.733	0.972
FAI	SDF-1	Inflammatory/endothelial	-0.273	0.061	0.398
AMH	1,25(OH)$_2$D$_3$	Vitamin D	-0.144	0.394	0.955

AMH	$25(OH)D_3$	Vitamin D	-0.094	0.526	0.955
AMH	$25(OH)D_2$	Vitamin D	-0.355	0.031	0.326
AMH	$24,25(OH)_2D_3$	Vitamin D	0.007	0.961	0.972
AMH	E-selectin	Inflammatory/endothelial	0.023	0.875	0.972
AMH	ICAM-1	Inflammatory/endothelial	-0.181	0.218	0.816
AMH	VCAM-1	Inflammatory/endothelial	-0.108	0.466	0.955
AMH	Cadherin-5	Inflammatory/endothelial	-0.046	0.754	0.972
AMH	P-selectin	Inflammatory/endothelial	0.010	0.944	0.972
AMH	von Willebrand factor	Inflammatory/endothelial	-0.028	0.851	0.972
AMH	ICAM-3	Inflammatory/endothelial	-0.072	0.626	0.955
AMH	ICAM-5	Inflammatory/endothelial	-0.107	0.470	0.955
AMH	ICAM-2	Inflammatory/endothelial	-0.142	0.337	0.936
AMH	VEGF-A	Inflammatory/endothelial	0.069	0.641	0.955
AMH	Angiopoietin-1	Inflammatory/endothelial	-0.038	0.796	0.972
AMH	Angiopoietin-2	Inflammatory/endothelial	0.080	0.587	0.955
AMH	Soluble Tie-2	Inflammatory/endothelial	0.124	0.400	0.955
AMH	TNF	Inflammatory/endothelial	0.093	0.528	0.955
AMH	IL-1α	Inflammatory/endothelial	-0.029	0.844	0.972
AMH	IL-6	Inflammatory/endothelial	0.045	0.761	0.972
AMH	Tissue Factor	Inflammatory/endothelial	-0.097	0.513	0.955
AMH	tPA	Inflammatory/endothelial	-0.159	0.280	0.899
AMH	PAI-1	Inflammatory/endothelial	-0.075	0.614	0.955
AMH	D-dimer	Inflammatory/endothelial	-0.014	0.926	0.972
AMH	SDF-1	Inflammatory/endothelial	-0.157	0.288	0.899
