# Supplementary material for: A Cross-Sectional Exploratory Study of Endothelial Biomarkers in Non-Obese Women with and Without Polyendocrine Metabolic Ovarian Syndrome
Source: Int J Mol Sci. 2026 Aug 26;27(17):7661. doi: 10.3390/ijms27177661 (PMC13566641; doi:10.3390/ijms27177661)
Supplement: Supplementary file 1 [file ijms-27-07661-s001.zip › Table S5 PMOS FAI HOMA AMH_aeb.pdf]

Supplementary Table S5. Complete Pearson correlation analyses of HOMA-IR, FAI and AMH with vitamin D metabolites and inflammatory/endothelial biomarkers in the PMOS cohort

Benjamini–Hochberg false-discovery-rate correction was applied within each predefined family of correlations. Pearson r denotes the Pearson correlation coefficient; p denotes the unadjusted two-sided p value; q denotes the BH-FDR-adjusted p value. q<0.05 was considered statistically significant.

Predictor	Outcome	Category	Pearson r	Pearson p	Pearson q (BH-FDR)
HOMA-IR	$1,25(OH)_2D_3$	Vitamin D	0.425	0.089	0.667
HOMA-IR	$25(OH)D_3$	Vitamin D	0.327	0.159	0.740
HOMA-IR	$25(OH)D_2$	Vitamin D	-0.024	0.937	0.963
HOMA-IR	$24,25(OH)_2D_3$	Vitamin D	0.319	0.170	0.740
HOMA-IR	E-selectin	Inflammatory/endothelial	0.230	0.329	0.740
HOMA-IR	ICAM-1	Inflammatory/endothelial	0.279	0.233	0.740
HOMA-IR	VCAM-1	Inflammatory/endothelial	-0.299	0.200	0.740
HOMA-IR	Cadherin-5	Inflammatory/endothelial	-0.239	0.310	0.740
HOMA-IR	P-selectin	Inflammatory/endothelial	0.246	0.295	0.740
HOMA-IR	von Willebrand factor	Inflammatory/endothelial	0.086	0.718	0.883
HOMA-IR	ICAM-3	Inflammatory/endothelial	0.010	0.966	0.979
HOMA-IR	ICAM-5	Inflammatory/endothelial	-0.457	0.043	0.399
HOMA-IR	ICAM-2	Inflammatory/endothelial	0.670	0.001	**0.046**
HOMA-IR	VEGF-A	Inflammatory/endothelial	-0.160	0.501	0.786
HOMA-IR	Angiopoietin-1	Inflammatory/endothelial	0.070	0.771	0.917
HOMA-IR	Angiopoietin-2	Inflammatory/endothelial	-0.282	0.228	0.740
HOMA-IR	Soluble Tie-2	Inflammatory/endothelial	-0.242	0.304	0.740
HOMA-IR	TNF	Inflammatory/endothelial	0.200	0.398	0.786
HOMA-IR	IL-1α	Inflammatory/endothelial	0.420	0.065	0.543
HOMA-IR	IL-6	Inflammatory/endothelial	-0.036	0.880	0.943
HOMA-IR	Tissue Factor	Inflammatory/endothelial	-0.469	0.037	0.399

HOMA-IR	tPA	Inflammatory/endothelial	0.321	0.168	0.740
HOMA-IR	PAI-1	Inflammatory/endothelial	0.164	0.489	0.786
HOMA-IR	D-dimer	Inflammatory/endothelial	0.228	0.334	0.740
HOMA-IR	SDF-1	Inflammatory/endothelial	-0.286	0.222	0.740
FAI	$1,25(OH)_2D_3$	Vitamin D	0.587	0.013	0.236
FAI	$25(OH)D_3$	Vitamin D	0.249	0.289	0.740
FAI	$25(OH)D_2$	Vitamin D	0.182	0.552	0.812
FAI	$24,25(OH)_2D_3$	Vitamin D	0.227	0.335	0.740
FAI	E-selectin	Inflammatory/endothelial	0.718	<0.001	**0.027**
FAI	ICAM-1	Inflammatory/endothelial	0.361	0.118	0.738
FAI	VCAM-1	Inflammatory/endothelial	0.138	0.563	0.812
FAI	Cadherin-5	Inflammatory/endothelial	0.159	0.503	0.786
FAI	P-selectin	Inflammatory/endothelial	0.101	0.671	0.868
FAI	von Willebrand factor	Inflammatory/endothelial	-0.221	0.350	0.749
FAI	ICAM-3	Inflammatory/endothelial	0.181	0.446	0.786
FAI	ICAM-5	Inflammatory/endothelial	0.199	0.401	0.786
FAI	ICAM-2	Inflammatory/endothelial	0.532	0.016	0.236
FAI	VEGF-A	Inflammatory/endothelial	-0.003	0.988	0.988
FAI	Angiopoietin-1	Inflammatory/endothelial	-0.169	0.477	0.786
FAI	Angiopoietin-2	Inflammatory/endothelial	0.237	0.313	0.740
FAI	Soluble Tie-2	Inflammatory/endothelial	-0.182	0.443	0.786
FAI	TNF	Inflammatory/endothelial	0.285	0.224	0.740
FAI	IL-1α	Inflammatory/endothelial	0.029	0.904	0.951
FAI	IL-6	Inflammatory/endothelial	-0.074	0.755	0.913
FAI	Tissue Factor	Inflammatory/endothelial	0.162	0.496	0.786
FAI	tPA	Inflammatory/endothelial	0.188	0.428	0.786
FAI	PAI-1	Inflammatory/endothelial	-0.119	0.617	0.843
FAI	D-dimer	Inflammatory/endothelial	-0.192	0.417	0.786
FAI	SDF-1	Inflammatory/endothelial	-0.368	0.111	0.738
AMH	$1,25(OH)_2D_3$	Vitamin D	-0.497	0.043	0.399

AMH	$25(OH)D_3$	Vitamin D	-0.255	0.279	0.740
AMH	$25(OH)D_2$	Vitamin D	-0.413	0.161	0.740
AMH	$24,25(OH)_2D_3$	Vitamin D	-0.162	0.496	0.786
AMH	E-selectin	Inflammatory/endothelial	0.041	0.864	0.939
AMH	ICAM-1	Inflammatory/endothelial	-0.259	0.270	0.740
AMH	VCAM-1	Inflammatory/endothelial	-0.341	0.141	0.740
AMH	Cadherin-5	Inflammatory/endothelial	-0.194	0.413	0.786
AMH	P-selectin	Inflammatory/endothelial	0.095	0.691	0.870
AMH	von Willebrand factor	Inflammatory/endothelial	-0.147	0.536	0.804
AMH	ICAM-3	Inflammatory/endothelial	-0.119	0.618	0.843
AMH	ICAM-5	Inflammatory/endothelial	0.109	0.649	0.854
AMH	ICAM-2	Inflammatory/endothelial	0.065	0.787	0.922
AMH	VEGF-A	Inflammatory/endothelial	-0.123	0.606	0.843
AMH	Angiopoietin-1	Inflammatory/endothelial	0.093	0.696	0.870
AMH	Angiopoietin-2	Inflammatory/endothelial	-0.259	0.271	0.740
AMH	Soluble Tie-2	Inflammatory/endothelial	0.114	0.632	0.847
AMH	TNF	Inflammatory/endothelial	0.048	0.840	0.939
AMH	IL-1α	Inflammatory/endothelial	0.026	0.913	0.951
AMH	IL-6	Inflammatory/endothelial	-0.059	0.803	0.927
AMH	Tissue Factor	Inflammatory/endothelial	-0.043	0.857	0.939
AMH	tPA	Inflammatory/endothelial	-0.578	0.008	0.191
AMH	PAI-1	Inflammatory/endothelial	-0.041	0.863	0.939
AMH	D-dimer	Inflammatory/endothelial	-0.151	0.526	0.804
AMH	SDF-1	Inflammatory/endothelial	-0.246	0.295	0.740
